# Diagnostik, Therapie und Verlaufskontrolle der diabetischen Augenerkrankung (Update 2026)

**DOI:** 10.1007/s00508-025-02691-8

**Published:** 2026-04-30

**Authors:** Andreas Pollreisz, Vanessa Gasser-Steiner, Bianca Gerendas, Stefan Mennel, Stephan Radda, Stefan Sacu, Ulrike Stolba, Andreas Wedrich

**Affiliations:** 1 Universitätsklinik für Augenheilkunde und Optometrie, Medizinische Universität Wien, Wien, Österreich; 2 Augenärztliche Ordination Gasser-Steiner, Weiz, Österreich; 3 Abteilung für Augenheilkunde, Landeskrankenhaus Feldkirch, Feldkirch, Österreich; 4 Abteilung für Augenheilkunde, Hanusch-Krankenhaus, Wien, Österreich; 5 Augenabteilung, Klinik Landstraße, Wien, Österreich; 6 Universitäts-Augenklinik, Medizinische Universität Graz, Graz, Österreich

**Keywords:** Diabetische Retinopathie, Diabetisches Makulaödem, Intravitreale operative Medikamentenapplikation, Retinale Laserkoagulation, Vitrektomie, Diabetic retinopathy, Diabetic macular edema, Intravitreal drug delivery, Retinal laser photocoagulation, Vitrectomy

## Abstract

Diabetes mellitus kann zu unterschiedlichen Augenerkrankungen führen wie diabetische Retinopathie, diabetisches Makulaödem, Optikusneuropathie, Katarakt, Sekundärglaukom und Fehlfunktionen der äußeren Augenmuskeln. Die Inzidenz dieser Spätschäden korreliert mit der Dauer des Diabetes und mit dem Grad der metabolischen Kontrolle. Reguläre augenfachärztliche Kontrollen sind notwendig, um visusbedrohende Spätschäden einer diabetischen Augenerkrankung frühzeitig zu erkennen und entsprechende Therapien einzuleiten.

## Abstract (en)

Diabetes mellitus can lead to various eye diseases, such as diabetic retinopathy, diabetic macular edema, optic neuropathy, cataract, secondary glaucoma and dysfunction of the eye muscles. The incidence of these delayed disorders correlates with the duration of diabetes and the degree of metabolic control. Regular ophthalmological control examinations are needed for early recognition of sight-threatening advanced stages of diabetic eye diseases and to initiate appropriate treatment.

## 1.1 Einleitung

Die vorliegenden Leitlinien wurden 2004 von einer Arbeitsgruppe der Netzhautkommission der Österreichischen Ophthalmologischen Gesellschaft zusammengestellt, im Jahr 2009, 2012, 2015, 2018 und 2022 überarbeitet und nun für die vorliegende Version entsprechend dem Stand der Wissenschaft vom Juni 2025 komplett aktualisiert. Als Grundlage für diese Leitlinien wurden neben der aktuellen Literatur vor allem die „Preferred Practice Pattern Diabetic Retinopathy“ der American Academy of Ophthalmology [1], Leitlinien der Deutschen Diabetesgesellschaft [2] sowie Empfehlungen der American Diabetes Association [3] verwendet.

## 1.2 Epidemiologie

Eine Retinopathie findet sich bei 24–27 % aller Menschen mit Typ-1-Diabetes und bei 9–16 % mit Typ-2-Diabetes, wobei in letzterer Gruppe bereits in 2–16 % aller Fälle diabetische Netzhautveränderungen bei Diagnosestellung vorhanden sind [2].

Neben der diabetischen Retinopathie (DRP) und Makulopathie (DMP) kann Diabetes mellitus weitere Spätschäden im Bereich des Auges wie Optikusneuropathie, Sekundärglaukom, Katarakt und Lähmungen der Augenmuskeln verursachen.

Die Inzidenz dieser Spätschäden korreliert einerseits mit der Diabetesdauer, andererseits mit der Qualität der metabolischen Kontrolle [4].

Die DRP/DMP ist neben dem Glaukom die häufigste Ursache für vollständige Erblindung in den entwickelten Ländern.

## 1.3 Biomarker für kardiovaskuläre Komplikationen

Basierend auf den „Guidelines on diabetes, pre-diabetes, and cardiovascular diseases“ der European Society of Cardiology (ESC) und der European Association for the Study of Diabetes (EASD), führt die Diagnose einer DRP/DMP bei Menschen mit Typ-2-Diabetes zu einer Einstufung in die „Very high risk“-Kategorie hinsichtlich kardiovaskulärer Ereignisse [5].

## 1.4 Risikofaktoren

Der wichtigste Risikofaktor für das Entstehen und die Progredienz einer DRP ist die chronische Hyperglykämie [6, 7].

Weitere Risikofaktoren sind arterielle Hypertonie, Diabetesdauer, Hyperlipidämie [6, 7], hormonelle Umstellungen in Pubertät [8] und Schwangerschaft [9] sowie abrupte Absenkung des HbA_1c_ durch Optimierung der Therapie (Beginn einer intensivierten Insulintherapie, Umstellung von oraler auf Insulintherapie, GLP-1-Rezeptoragonisten) [10, 11, 39, 40].

Für das Auftreten eines diabetischen Makulaödems (DMÖ) ist neben der chronischen Hyperglykämie vor allem die arterielle Hypertonie ein signifikanter Risikofaktor [12].

### Frühverschlechterung der DRP

Eine Frühverschlechterung („early worsening“, „euglycemic reentry“) der Retinopathie kann in seltenen Fällen bei Typ-1- und Typ-2-Diabetes auftreten (< 5 %). Sie kommt meist innerhalb von 12 Monaten nach einer Stoffwechselverbesserung (z. B. durch intensivierte Insulintherapie, GLP-1-Rezeptoragonisten oder bariatrische Chirurgie) vor. Risikofaktoren sind lange Diabetesdauer (> 10 Jahre), schlechte Stoffwechseleinstellung (HbA_1c_-Wert > 10 %) und vorbestehende Retinopathie. Bei einer geplanten rapiden HbA_1c_-Senkung und Vorliegen dieser Risikofaktoren ist in den ersten 12 Monaten eine 3‑monatige augenfachärztliche Kontrolle empfohlen. Eine graduelle HbA_1c_-Senkung zur Risikominimierung wird kontrovers diskutiert. Langfristig überwiegt jedoch der Nutzen der Blutzuckerverbesserung.

### Spezialfall GLP-1-Rezeptoragonisten

Eine sehr seltene Nebenwirkung von Semaglutid (bis zu 1 von 10.000 Behandelte) ist die nichtarteriitische anteriore ischämische Optikusneuropathie (NAION), eine akute Durchblutungsstörung des Sehnervenkopfes mit plötzlichem, schmerzlosem Sehverlust. Bei plötzlicher oder rascher Verschlechterung der Sehfähigkeit ist umgehend augenfachärztlicher Rat einzuholen. Bei bestätigter NAION ist Semaglutid umgehend abzusetzen [41]. Ebenfalls sehr selten ist ein erhöhtes Risiko für eine neovaskuläre altersbedingte Makuladegeneration (AMD), die zu einer Sehbeeinträchtigung führt und augenfachärztlicher Abklärung und Behandlung bedarf [42]. Angesichts der Seltenheit dieser Nebenwirkungen von GLP-1-Rezeptoragonisten ist das Nutzen-Risiko-Verhältnis jedoch als deutlich positiv zu bewerten.

## 1.5 Diagnostik

Jede augenfachärztliche Kontrolle umfasst folgende Basisuntersuchungen:

- bestkorrigierte Sehschärfe/Visus,
- Augendruck,
- Biomikroskopie vorderer und hinterer Augenabschnitt mit erweiterter Pupille.

Folgende weitere Untersuchungsmethoden werden je nach Netzhautbefund durchgeführt:

- Optische Kohärenztomographie („optical coherence tomography“ [OCT])
  Eine OCT-Untersuchung ermöglicht nichtinvasiv die Visualisierung einzelner Netzhautschichten und erlaubt somit eine detaillierte dreidimensionale Darstellung und Lokalisierung eines Netzhautödems, eine Beurteilung der Integrität der Photorezeptoren, eine Bestimmung der Dicke der Nervenfaserschichten sowie eine Darstellung der Interaktionen zwischen Netzhaut und Glaskörpergrenzschicht [13].
- OCT-Angiographie (OCTA)
  Dies stellt eine funktionelle Erweiterung der konventionellen OCT-Technologie dar und erlaubt die nichtinvasive Messung von Blutfluss in den Gefäßen der Netzhaut, Choriokapillaris und Aderhaut innerhalb von Sekunden. Mit dieser Untersuchung können nicht perfundierte Netzhautareale detektiert sowie Neovaskularisationen und andere mikrovaskuläre Veränderungen diagnostiziert werden [14–16].
- Fluoreszenzangiographie (FLA)
  Nach intravenöser Verabreichung von Fluoreszein wird mittels einer speziellen Funduskamera innerhalb von Sekunden bis Minuten die Verteilung des Farbstoffes in den Netzhautgefäßen visualisiert, womit Ischämien und Neovaskularisationen (Leckagen) detektiert werden können [17].
- Digitale Farbfotografie der Netzhaut
  Mittels einer Funduskamera werden Fotoaufnahmen der Netzhaut angefertigt, die v. a. für Verlaufskontrollen einen wichtigen Stellenwert haben [18].
- B‑Scan-Sonographie
  Untersuchung der Netzhaut bei fehlendem Funduseinblick, wie z. B. bei Glaskörperblutung oder dichter Katarakt.

## 1.6 Stadien der diabetischen Retinopathie

Die DRP ist eine Mikroangiopathie charakterisiert durch Degeneration von Perizyten, gesteigerter Permeabilität der Gefäße und Kapillarokklusion [19]. Eine Einteilung kann nach der „International Clinical Diabetic Retinopathy Disease Severity Scale“ in eine nichtproliferative diabetische Retinopathie (NPDRP) und proliferative diabetische Retinopathie (PDRP) erfolgen, wobei die NPDRP aufgeteilt wird in die Stadien mild, mäßig und schwer [1].

### 1.6.1 Nichtproliferative diabetische Retinopathie (NPDRP)

Die einzelnen Stadien sind charakterisiert durch folgende Netzhautveränderungen:

- *mild: *einzelne Mikroaneurysmen,
- *mäßig: *weniger als 20 Mikroaneurysmen und intraretinale Blutungen pro Netzhautquadrant, perlschnurartige Veränderungen an den Venen in maximal 1 Quadranten,
- *schwer:* mehr als 20 Mikroaneurysmen und intraretinale Blutungen in jedem der 4 Netzhautquadranten, perlschnurartige Venenveränderungen in mindestens 2 Quadranten oder intraretinale mikrovaskuläre Anomalien (IRMA – Kapillarbettveränderungen) in mindestens 1 Quadranten („4-2-1“ Regel).

### 1.6.2 Proliferative diabetische Retinopathie (PDRP)

Folgende Netzhautveränderungen und Komplikationen können auftreten:

- Neovaskularisationen im Bereich der Papille oder an anderen Netzhautstellen,
- Glaskörperblutung, präretinale Blutung aus qualitativ minderwertigen Neovaskularisationen,
- Fibrosierung der Neovaskularisationen,
- Netzhautablösung durch Kontraktion von fibrosierenden Neovaskularisationen (Traktionsamotio),
- Sekundärglaukom durch Neovaskularisationen im vorderen Augenabschnitt (Kammerwinkel).

### 1.6.3 Diabetisches Makulaödem (DMÖ)

Ein DMÖ kann sich in jedem Stadium der DRP zeigen, und es tritt bei Menschen mit Typ-1-Diabetes häufiger als bei Menschen mit Typ-2-Diabetes auf (15 % vs. 6 %) [1, 2].

Es wird zwischen einem visusmindernden und einem Makulaödem ohne Visusverlust unterschieden, was Konsequenzen für die augenfachärztliche Therapieentscheidung hat. Therapiert wird basierend auf Sehschärfe sowie Ausmaß und Ausbreitung des Ödems in Richtung Fovea (Stelle des schärfsten Sehens in der Netzhautmitte) [20].

## 1.7 Untersuchungszeitpunkte für Screening und im Verlauf

Eine augenfachärztliche Untersuchung soll zeitnah zum Zeitpunkt der Diabetes-Diagnosestellung bei Menschen mit Typ-2-Diabetes und ab dem 11. Lebensjahr bei Menschen mit Typ-1-Diabetes erfolgen.

Bei bisher nicht diagnostizierter DRP sowie Vorliegen mindestens eines internistischen Risikofaktors (lange Diabetesdauer, erhöhter HbA_1c_-Wert, Hypertonie, Nephropathie) oder bei unbekanntem medizinischem Risiko (keine rezente internistische/allgemeinmedizinische Untersuchung) ist eine jährliche Verlaufskontrolle notwendig [1, 2].

Sind bereits diabetische Netzhautveränderungen bekannt, erfolgen die augenfachärztlichen Untersuchungen entsprechend dem Schweregrad der DRP:

- *milde nichtproliferative diabetische Retinopathie: *jährlich,
- *mäßige nichtproliferative diabetische Retinopathie: *halbjährlich,
- *schwere nichtproliferative diabetische Retinopathie:* vierteljährlich,
- *proliferative diabetische Retinopathie:* laufende augenärztliche Kontrolle,
- *diabetisches Makulaödem:* laufende augenärztliche Kontrolle.

### 1.7.1 Augenfachärztliche Untersuchungsintervalle bei Spezialfällen

- Kinder mit Typ-1-Diabetes vor dem 11. Lebensjahr
  Ab einer Diabetesdauer von 5 Jahren
- Kinder mit Typ-1-Diabetes ab dem 11. Lebensjahr
  Zum Zeitpunkt der Diabetes-Diagnosestellung
- Schwangere mit Diabetes mellitus
  Bei Feststellung der Schwangerschaft mit Verlaufskontrollen alle 3 Monate
- Neueinstellung oder Therapieintensivierung des Blutzuckers
  Zeitnahe und regelmäßige Untersuchungen bei Gefahr des Neuauftretens oder Progression der DRP
- Therapieintensivierung mit GLP-1-Rezeptoragonisten oder Insulin: Untersuchung vor Medikamentengabe bei Gefahr der Verschlechterung der DRP (Deutsche Diabetes Gesellschaft, PE Diabetische Retinopathie, Stand: 11/2024)
- Bariatrischer Eingriff
  Untersuchung vor geplanter OP [21]

## 1.8 Interdisziplinäre allgemeine ophthalmologische Behandlungsziele

Interdisziplinäres ophthalmologisches Ziel zur Behandlung von Menschen mit Diabetes mellitus ist die Verhinderung des Auftretens einer DRP sowie schlussendlich die Vermeidung von Sehverlust und Erblindung durch optimale Einstellung von Blutzucker, Blutdruck und Blutfetten sowie der Verzicht auf Rauchen in Verbindung mit einer rechtzeitigen adäquaten ophthalmologischen Therapie [22].

## 1.9 Ophthalmologische Behandlungsmöglichkeiten der DRP

1. Intravitreale operative Medikamentenapplikation (IVOM) von Antikörpern (alphabetische Auflistung nach Wirkstoffbezeichnung): Aflibercept 2 mg – Eylea®, Aflibercept 8 mg – Eylea® 8 mg, Bevacizumab – Avastin® („off label“), Brolucizumab – Beovu®, Faricimab – Vabysmo®, Ranibizumab – Lucentis®
2. IVOM von Steroiden: Triamcinolon, Kortisonimplantat (Dexamethason – Ozurdex®, Fluocinolonacetonid – Iluvien®)
3. Fokale und/oder gitterförmige Laserkoagulation
4. Panretinale Laserkoagulation („mild scatter“, voll)
5. Pars-plana-Vitrektomie (Glaskörperentfernung mit/ohne Membranentfernung und evtl. Endotamponade mit Silikonöl oder Gas)

## 1.10 Therapieoptionen der einzelnen DRP-Stadien [3, 20, 23]

### 1.10.1 Mäßige bis schwere NPDR

Die „DRCR Retina Network“-Studie „Protokoll W“ untersuchte einen möglichen prophylaktischen Effekt einer Anti-VEGF-IVOM-Therapie bei Patient:innen mit mäßiger bis schwerer NPDR hinsichtlich Auftretens visusbedrohender Komplikationen wie DMÖ oder PDRP sowie Sehleistung. Im Beobachtungszeitraum von 2 Jahren konnte zwar eine niedrigere Rate an visusbedrohenden Komplikationen gezeigt werden, dies hatte jedoch keinen Benefit auf den Visus, wenn Patienten erst beim Auftreten der Komplikationen mittels Anti-VEGF-IVOM behandelt wurden [24].

Bei Vorhandensein von ausgedehnten Netzhautischämien sowie eingeschränkter Compliance bei erhöhtem internistischem Risikoprofil ist eine partielle/komplette panretinale Laserkoagulation in Erwägung zu ziehen.

### 1.10.2 Proliferative DRP (PDRP)

Primäre Behandlung einer PDRP ohne Glaskörperblutung und Netzhautabhebung ist eine komplette panretinale Laserbehandlung. Die „DRCR Retina Network“-Studie „Protokoll S“ hat innerhalb einer Follow-up-Zeit von 5 Jahren gezeigt, dass eine intravitreale Anti-VEGF-Therapie der panretinalen Laserkoagulation hinsichtlich Visus nicht unterlegen ist [25]. Es hat sich jedoch in dieser Studie gezeigt, dass Patienten in der Anti-VEGF-IVOM-Gruppe kontinuierlich intravitreale Injektionen über den gesamten Nachbeobachtungszeitraum benötigen (durchschnittlich 19 Applikationen in 5 Jahren). Weiters wurde beobachtet, dass Patienten ohne panretinale Laserkoagulation bei Nichteinhalten der empfohlenen Injektionstherapien und Kontrolluntersuchungen ein sehr hohes Risiko eines massiven Visusverlustes haben [26].

### 1.10.3 Diabetisches Makulaödem (DMÖ)

Randomisierte klinische Studien haben gezeigt, dass eine Anti-VEGF-IVOM-Therapie die bevorzugte Behandlung bei einem DMÖ mit Foveabeteiligung und entsprechenden subjektiven Sehbeschwerden ist [27–31]. In der „DRCR Retina Network“-Studie „Protokoll V“ wurde gezeigt, dass Patienten mit einem foveabeteiligenden DMÖ und gutem Visus (≥ 0,8 gemessen mittels „ETDRS“-Tafeln) ohne Behandlung keine schlechtere Sehleistung nach 2 Jahren zeigten als Patienten die entweder mit Anti-VEGF-IVOM oder Laserkoagulation behandelt wurden [32]. Für die Behandlung des DMÖs stehen aktuell (Stand Oktober 2022) insgesamt 5 verschiedene Medikamente in Österreich zur Verfügung, wobei Bevacizumab, Brolucizumab und Ranibizumab gegen VEGF‑A gerichtet sind und Aflibercept 2 mg und Aflibercept 8 mg zusätzlich VEGF‑B und „placental growth factor“ als Ziel hat. Faricimab ist ein bispezifischer Antikörper, der unabhängig VEGF‑A und Angiopoetin‑2 bindet [33].

Wenn ein unzureichendes funktionelles und morphologisches Ansprechen auf Anti-VEGF-IVOMs besteht, kann auf intravitreale Steroide gewechselt werden, wobei eine IVOM von Triamcinolon oder von Implantaten (Dexamethason, Fluocinolonacetonid) zur Verfügung steht [1]. Hier gilt zu beachten, dass Steroide zu Nebenwirkungen wie Katarakt oder Glaukom führen können.

Bei einem Ödem, das die Fovea noch nicht betrifft, jedoch eine Progression dahingehend zeigt, kann eine „Focal-grid“-Laserkoagulation erfolgen. Hierbei werden entweder Mikroaneurysmen oder Blutungen in diesem Areal („focal“) oder Bereiche mit Ödem („grid“) gelasert [34].

### 1.10.4 Fortgeschrittene diabetische Augenerkrankung

Treten als Folge der diabetischen Retinopathie Komplikationen wie Netzhautablösung, Glaskörperblutung und/oder eine Rubeosis iridis auf, spricht man von einer fortgeschrittenen diabetischen Augenerkrankung. Neben Vitrektomie inklusive Entfernung eventuell vorhandener Netzhautmembranen und -traktionen und panretinaler Laserkoagulation (Netzhautablösung, Glaskörperblutung), sowie Anti-VEGF-IVOM (Glaskörperblutung [35, 36]) können bei Sekundärglaukom auch zyklodestruktive (= den Ziliarkörper verödende) Verfahren wie Zyklophotokoagulation oder Implantation eines drucksenkenden Drains indiziert sein.

### 1.10.5 Milde bis mäßige nichtproliferative diabetische Retinopathie

- Observanz

### 1.10.6 Schwere nichtproliferative diabetische Retinopathie

- Milde/volle panretinale Laserkoagulation unter bestimmten Konditionen
  (ausgedehnte Netzhautischämien; eingeschränkte Compliance; erhöhtes internistisches Risikoprofil wie Typ-1-Diabetes, arterielle Hypertonie)

### 1.10.7 Proliferative diabetische Retinopathie

- Volle panretinale Laserkoagulation
- Glaskörper‑/Netzhautchirurgie
- Anti-VEGF-IVOM

### 1.10.8 Diabetisches Makulaödem

Basierend auf Sehschärfe sowie Lokalisation und Ausmaß des DMÖs:

- Anti-VEGF-IVOM
- IVOM von Steroiden
- fokale/gitterförmige Laserkoagulation

### 1.10.9 Glaskörpertraktion bei diabetischem Makulaödem

- Optional Vitrektomie

### 1.10.10 Glaskörperblutung

- Observanz und/oder Anti-VEGF-IVOM (nach Sonographie zum Ausschluss einer Netzhautabhebung)
- Vitrektomie mit panretinaler Laserkoagulation

### 1.10.11 Fibrosierte Neovaskularisationen mit Netzhauttraktion

- Vitrektomie mit Peeling der fibrovaskulären Membranen und panretinaler Laserkoagulation
